# Polycystins and cellular Ca^2+^ signaling

**DOI:** 10.1007/s00018-012-1188-x

**Published:** 2012-10-18

**Authors:** D. Mekahli, Jan B. Parys, G. Bultynck, L. Missiaen, H. De Smedt

**Affiliations:** Laboratory of Molecular and Cellular Signaling, Department of Cellular and Molecular Medicine, KU Leuven, Campus Gasthuisberg O/N-I, B-802, Herestraat 49, 3000 Leuven, Belgium

**Keywords:** Calcium signaling, Polycystin, ADPKD, Renal pathology

## Abstract

The cystic phenotype in autosomal dominant polycystic kidney disease is characterized by a profound dysfunction of many cellular signaling patterns, ultimately leading to an increase in both cell proliferation and apoptotic cell death. Disturbance of normal cellular Ca^2+^ signaling seems to be a primary event and is clearly involved in many pathways that may lead to both types of cellular responses. In this review, we summarize the current knowledge about the molecular and functional interactions between polycystins and multiple components of the cellular Ca^2+^-signaling machinery. In addition, we discuss the relevant downstream responses of the changed Ca^2+^ signaling that ultimately lead to increased proliferation and increased apoptosis as observed in many cystic cell types.

## Introduction

Autosomal dominant polycystic kidney disease (ADPKD) affects more than 1 in 1,000 live births and is the most common monogenic cause of kidney failure in humans [1–4]. ADPKD is characterized by the progressive formation and enlargement of renal cysts, typically leading to chronic renal failure by late middle age. In most cases, the disease arises as a consequence of mutations in the *PKD1* or *PKD2* genes, which encode the proteins polycystin-1 and -2, respectively. Mutations in the *PKD1* gene account for approximately 85 % (ADPKD type 1), and mutations in the *PKD2* gene account for approximately 15 % (ADPKD type 2) of the affected individuals [2]. Disease progression is typically more rapid in ADPKD type 1, with a mean age of end-stage renal disease approximately 20 years earlier than in type 2, but in all other respects ADPKD types 1 and 2 share almost identical disease phenotypes. This suggests that polycystin-1 and -2 function in common pathways, implying that loss of activity of either protein results in a very similar disease manifestation [5]. The biological role of the polycystin proteins and the molecular basis by which mutational malfunction of either of them leads to cystogenesis, have proven to be very complex, and have been discussed in several recent reviews [1, 2, 6–13]. A widely accepted view is that polycystin-1 and -2 are functionally associated in a receptor-ion channel complex, in which polycystin-1 acts as a receptor that gates the Ca^2+^-permeable polycystin-2 channel [14, 15]. Polycystin-1 (4,302 amino acids) contains a large extracellular N-terminal domain, 11 predicted transmembrane spanning segments, and an intracellular C-terminus [16]. The extracellular region of polycystin-1 contains >3,000 amino acids and is implicated in cell–cell and cell–matrix interactions. Polycystin-1 is cleaved at its predicted G-protein-coupled receptor proteolytic site, a feature that could be essential for its biological activity [17]. The intracellular C-terminus of polycystin-1 contains a coiled-coil domain that is involved in the physical interaction with other proteins, and in particular with polycystin-2 [18, 19]. Polycystin-2 is a smaller transmembrane protein (968 amino acids) predicted to have six transmembrane regions and sharing significant homology with transient receptor potential (TRP) channels [9, 12, 13, 20]. Better understanding of the role of the polycystin-1/polycystin-2 complex came from the observation that this co-assembly produced cation-permeable currents at the plasma membrane [21], and participated in mechano-sensation and flow-dependent Ca^2+^ signaling in the primary cilium [22]. As reviewed recently, there is a clear connection between polycystic kidney disease and dysfunction of ciliary proteins [13]. The precise cellular function of the polycystin proteins is, however, still not completely understood, particularly as both polycystins have been found in cellular locations other than the cilium [23]. Polycystin-1 has been localized to cell–cell junctions and both apical and basolateral membranes [23, 24]. Polycystin-2 is a resident endoplasmic-reticulum (ER) protein [25] and its trafficking is highly regulated [26–29]. The differential localization of both polycystins also suggests that these proteins may display different cellular functions either alone or as a protein complex [29, 30]. Several cellular mechanisms have been proposed to explain cyst formation and cyst growth including a change in cell polarity [31], an altered matrix composition [32], and remarkably, a disturbed balance between cell proliferation and apoptosis [33]. The view that polycystin-2 is a potential Ca^2+^ channel and polycystin-1 is a receptor regulating its activity, suggests that intracellular Ca^2+^ signaling could be one of the most proximal events in many cellular functions of the polycystins and consequently in the dysfunctional mechanisms that may lead to cyst formation. Clearly, the Ca^2+^ effects are not limited to the restricted compartment of the cilium but will also involve Ca^2+^ influx from other parts of the plasma membrane as well as Ca^2+^ release from the ER. The situation becomes even more complex as polycystin-2 was found to associate with other Ca^2+^ channels in the plasma membrane (TRPC1 [34, 35] and TRPV4 [36]), and in intracellular membranes (inositol 1,4,5-trisphosphate receptor (IP_3_R) [37, 38] and ryanodine receptor (RyR) [39]). Moreover, polycystin-1 has been found to interact with basic components of the Ca^2+^ toolkit such as the IP_3_R [40] and the stromal interaction molecule-1 (STIM1) [41]. Hence, polycystins may affect Ca^2+^ signaling in many different ways, including effects on cytosolic or ER Ca^2+^ concentration, global or local Ca^2+^ changes, Ca^2+^ oscillations, intracellular Ca^2+^-leak pathways or plasma-membrane Ca^2+^ influx or a combination of these effects. However, the cellular role of polycystins in Ca^2+^ signaling, and the downstream parameters that may link the disturbed Ca^2+^ signaling in ADPKD to cyst formation, are not yet understood [42]. In this review, we provide an update of the different effects of polycystins on cellular Ca^2+^ signaling. We also discuss the current view on the downstream signaling pathways that could be affected by the dysfunctional Ca^2+^ signals in ADPKD, ultimately leading to a cystic phenotype with increased proliferation and increased apoptosis.

## Disturbed cellular Ca^2+^ fluxes in ADPKD

### Cilium and plasma membrane

**Fig. 1:**
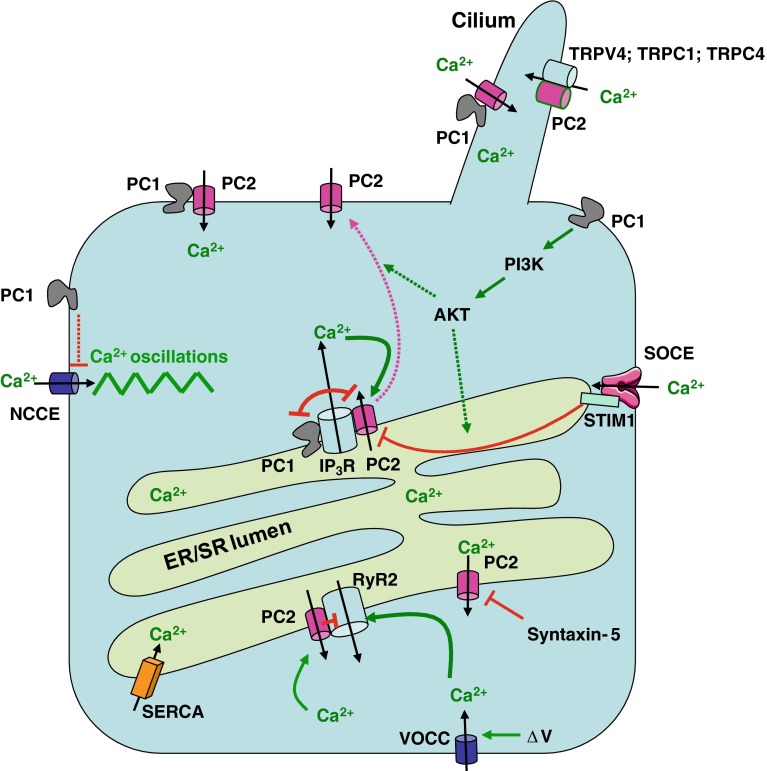
Major players controlling cellular Ca^2+^ signaling by polycystins. Polycystin-1 (PC1) and polycystin-2 (PC2) form a signaling complex in the cilium that mediates Ca^2+^ influx via PC2, possibly in response to mechanical stimuli. Also TRPV4, TRPC1, and TRPC4 interact with PC2 and could play a role in mechano-sensitive Ca^2+^ influx. PC2 is also present in the ER where it directly interacts with the IP_3_R and in cardiac cells also with the RyR2. PC2 behaves as a Ca^2+^-induced Ca^2+^-release channel and thereby amplifies IP_3_-induced Ca^2+^ release. The RyR2 is activated by Ca^2+^ influx via voltage-operated Ca^2+^ channels and is inhibited by PC2. Ca^2+^ leak via PC2 may be controlled by other proteins such as syntaxin-5. PC1 activates the PI3-K/AKT signaling. This leads (by as-yet-unresolved mechanisms) to an increase in the STIM1-IP_3_R interaction, which reduces the interaction between the IP_3_R and PC2 with possibly a translocation of PC2 to the plasma membrane. PC1 and PC2 compete for the same binding site on the IP_3_R. PC1 dysfunction leads to strengthening of the IP_3_R-PC2 interaction and remodeling of the Ca^2+^ fluxes with an increase of IICR, more ER Ca^2+^ depletion, and Ca^2+^ influx via activation of SOCE. PC1 also negatively modulates agonist-evoked NCCE activity through a still undefined mechanism. Loss of function of PC1 causes an increase in NCCE-channel activity leading to Ca^2+^ oscillations. *PC1/PC2* polycystin-1/-2, *NCCE* non-capacitive Ca^2+^ entry, *ΔV* voltage change over the plasma membrane, *VOCC* voltage-operated Ca^2+^ channel. Inhibitory and stimulatory mechanisms are represented by *red* and *green arrows*, respectively; the *purple arrow* represents the trafficking of PC2; *dotted lines* indicate that the mechanisms are as yet undefined

Polycystin-1 and -2 can form heteromeric complexes in vivo [43]. Importantly, co-expression of both proteins in Chinese hamster ovary (CHO) cells promoted the translocation of polycystin-2 to the plasma membrane and the complex produced a Ca^2+^-permeable non-selective cation channel [21]. Neither of the polycystins alone produced an ion current, while disease-associated mutants that are incapable of heterodimerization did not result in channel activity. Heterologous expression of both proteins resulted in the formation of a plasmalemmal ion-channel complex in neurons as well as in kidney cells, in which polycystin-2 activation occurred through structural rearrangement of polycystin-1 [14]. An important finding was that both proteins co-localize in the primary cilia of epithelial cells, where their role could be to promote mechano-sensation and fluid-flow sensation [22, 44] (Fig. 1). Cells isolated from transgenic mice that lack functional polycystin-1 formed cilia, but did not increase Ca^2+^ influx in response to physiological fluid flow. Inhibitory antibodies directed against polycystin-2 similarly abolished the flow response in wild-type cells. Defects in proteins involved in the function or structure of primary cilia such as cystin, polaris, inversin, and kinesin-II also cause polycystic kidney diseases [45]. Fluid shear-force bending of the cilium causes the influx of Ca^2+^ through mechanically sensitive channels in the ciliary membrane [46]. The Ca^2+^ signal could then be further amplified by Ca^2+^ release from IP_3_Rs or RyRs via a Ca^2+^-induced Ca^2+^-release (CICR) mechanism. This view proposes a dysregulated Ca^2+^ influx as an important first step in the initiation of cystogenesis [47].

There has been some confusion regarding the structural model for the polycystin-1/-2 complex. A newly identified coiled-coil domain in the C-terminus of polycystin-2 (a.a. 839–873), different from a more upstream coiled-coil domain (a.a. 772–796) [19], has been proposed to mediate assembly into a homotrimer to which a single coiled-coil domain in the C-terminus of polycystin-1 (a.a. 4214–4248) can bind [48, 49]. Other evidence, obtained by atomic force microscopy, however, showed that the polycystin-1/-2 complex assembles as a tetramer with a 2:2 stoichiometry [50]. The latter is more in line with recently described homo- and heteromeric polycystin-2 channel properties suggesting fourfold symmetry [35, 36, 51]. Differences between both models may be due to different structural properties of the helix containing a coiled-coil-domain motif, which may oligomerize differently as an isolated peptide than when embedded in the folded protein [52]. Deletion of this polycystin-2 coiled-coil domain (referred to as CC2 domain) indicated that although this domain is required for heterotypic interaction with polycystin-1, it does not represent the binding site itself [52]. In agreement with earlier studies [19, 48], the domain responsible for binding was found distal from CC2 (a.a. 872–895). Furthermore, there is evidence for a dimerization site in polycystin-2, N-terminally located of the first transmembrane domain, which regulates channel tetramerization [53]. Although CC2 is considered an assembly domain, it does not seem to have a prominent role in the self-association of polycystin-2 [52]. Polycystin-2 channels with CC2 deletions still tetramerize [52], and C-terminal mutants can co-immunoprecipitate full-length polycystin-2 [53]. The role of the C-terminus of polycystin-2 may therefore be to provide an essential scaffolding platform for heteromeric assembly with other channel proteins, including polycystin-1 [19], TRPC1 [34], TRPV4 [36], and the IP_3_R [37].

The polycystin-2 C-terminus is important for the regulation of the Ca^2+^-channel activity [54–56]. An EF-hand motif was identified connected by a linker to a coiled-coil domain overlapping with CC2 [54]. An affinity for Ca^2+^ in the micromolar range was found for the EF-hand domain by isothermal titration calorimetry. This region may therefore sense local Ca^2+^ concentration changes and operate as a Ca^2+^-sensitive switch with a role in proper folding and oligomerization of polycystin-2 [54] and subsequent channel gating [56].

Polycystin-2 can form spontaneously active nonselective cation channels in lipid bilayers [35, 57, 58]. Analysis of the channel properties revealed a high-conductance, nonselective, voltage-dependent cation channel [58]. Using various organic cations of different size, the pore diameter was estimated to be at least 1.1 nm [59]. Heterologous expression in *Xenopus* oocytes revealed a channel that is sensitive to changes of the cytosolic Ca^2+^ concentration [60]. Spontaneous activity of polycystin-2 was, however, not always obtained upon heterologous expression of polycystin-2 and polycystin-1 [48], which clearly illustrates the difficulty in identifying the physiological activation mechanisms of polycystin-2 or of the polycystin-1/-2 complex. An even larger complexity is suggested from the observations that polycystin-2 may associate and perhaps form heteromeric channels with other TRP-family members such as TRPC1 [34, 35, 51], TRPC4 [61], and TRPV4 [36] (Fig. 1). The function and regulation of polycystin-2 at the plasma membrane downstream of mechanical stimulation, cell-surface receptors, and cell adhesion, were recently discussed in an excellent review [12]. A role for polycystin-2 as a mechanosensitive channel has been suggested from measurements of changes in the cytosolic Ca^2+^ concentration in response to fluid flow [22, 62–64]. Also, regulation of polycystin-2-channel activity by environmental signals such as hydrostatic and osmotic pressure [65] and by cytoskeletal [66] and microtubular [67] elements in the human syncytiotrophoblast supports such a role. Mechanosensitivity of polycystin-2 may result from its interaction with TRPV4, which was required for fluid flow-induced Ca^2+^ entry in Madin–Darby canine kidney (MDCK) cells [36]. Loss of TRPV4, however, did not result in cyst formation, which suggests that mechanosensitive activation of this channel complex alone is not sufficient for cyst formation [36]. Polycystin-2 can also function as a bona fide receptor-operated channel downstream of epidermal growth factor (EGF) receptor activation [68]. EGF-induced activation of polycystin-2 required the activation of phospholipase C (PLC). Polycystin-2 interacted with PLC-γ2 and colocalized in the primary cilium with the EGF receptor and phosphatidyl inositol 4,5-bisphosphate (PIP_2_) [68]. EGF-induced activation of PIP_2_ breakdown resulted in the relieve of PIP_2_-mediated inhibition of polycystin-2 [68]. The localization of the EGF receptor in the primary cilium could represent a sensitization of the polycystin-2-channel activity with implications for cilium-based mechano-transduction, as it may reduce its threshold for activation by mechanical stimulation [12]. Channel activity of polycystin-2 complexed with TRPC1 but not with polycystin-1 could also be activated in response to PLC-coupled bradykinin-receptor stimulation [51]. This polycystin-2/TRPC1 channel with distinct properties from the polycystin-1/-2 complex has implications in mechano-sensation and cilium-based Ca^2+^ signaling [51]. Homologs of polycystin-1 and -2 have been shown to form receptor channel complexes acting as sour-taste receptors [69]. As there is increasing evidence from several gene-inactivation studies indicating that cilium-mediated mechano-transduction is not alone responsible for cyst formation, receptor-operated activation may play an important role [36, 51, 70–72]. A combined mechanism of flow-dependent delivery of a ligand would thereby become an intriguing possibility [13].

### Intracellular membranes

The intracellular distribution of polycystin-2 is very complex, with the largest pool in the ER in addition to its localization at the plasma membrane and in more restricted domains such as the primary cilium and mitotic spindles as discussed in several reviews [3, 27, 73]. Next to its role as a plasma-membrane Ca^2+^ channel and its ciliary function in complex with polycystin-1, polycystin-2 was proposed to have a function in intracellular Ca^2+^ release [25, 58]. Other findings, however, suggested that whereas heterologous expression of polycystin-2 showed a predominant ER localization, endogenous polycystin-2 was found primarily in the cilium and plasma membrane of mouse inner medullary collecting duct (IMCD) cells and in MDCK cells [74]. The long-standing controversy about this differential distribution has been clarified to some extent by the identification of specific signal sequences and trafficking proteins [3, 30, 60, 75]. A stretch of acidic amino acids in the C-terminus of polycystin-2 functions as an ER-retention signal by binding phosphofurin acidic cluster-sorting proteins (PACS-1 and -2) [25, 28]. Binding of PACS-1 and PACS-2 requires polycystin-2 phosphorylation by casein kinase II (CK-II) at Ser 812, and mediates retrieval back to the trans-Golgi network (PACS-1) and the ER (PACS-2), respectively [28]. Prevention of this phosphorylation in the *Caenorhabditis elegans* polycystin-2 homologue promoted its translocation to the cilium [76]. Polycystin-2 interactor Golgi- and ER-associated protein (PIGEA-14) is another regulator of polycystin-2 trafficking, causing its movement to a putative trans-Golgi compartment [77]. Plasma-membrane, but not cilia, localization of polycystin-2 is regulated by glycogen synthase kinase 3 (GSK3) phosphorylation of Ser 76 in the N-terminus [78]. In the presence of specific GSK3 inhibitors, the lateral plasma-membrane pool of endogenous polycystin-2 redistributes into an intracellular compartment in MDCK cells without any change in primary-cilia localization [78]. Furthermore, the N-terminus of polycystin-2 contains a motif (R6V7xP8), which is required for localization in the cilia [79]. Cyst cells expressing an ADPKD-associated polycystin-1 mutant had decreased amounts of both polycystin-1 and -2 in the primary cilium, indicating that impairing the function of one protein negatively affects the localization of the other [80].

An interaction between the C-termini of polycystin-1 and polycystin-2 is considered to be important for activation of the Ca^2+^-channel activity [14, 21]. This does not necessary require a co-localization in the same membrane, and a model for interaction with polycystin-2 either localized in the plasma membrane or in the ER has been proposed [47, 81]. The concept that polycystin-2 may be a novel type of intracellular Ca^2+^-release channel was based on the observation that polycystin-2 exogenously expressed in LLC-PK_1_ epithelial cells caused a marked augmentation of intracellular Ca^2+^ release upon vasopressin stimulation [58]. A similar role as an intracellular Ca^2+^-release channel was also found for the endogenous homologue of polycystin-2 in *Caenorhabditis elegans* [82]. The open probability of the channel was increased by Ca^2+^ in the physiological range (0.1–10 μM), whereas higher cytosolic [Ca^2+^] lowered the open probability [58]. The observation that polycystin-2 may function as a CICR channel was further strengthened by the sensitization towards Ca^2+^ upon CK-II phosphorylation at the C-terminal S812 site [83]. Polycystin-2-mediated Ca^2+^ release from the ER required activation of the IP_3_R [37, 58]. Moreover, it was demonstrated that polycystin-2 and the IP_3_R physically interact and the C-terminus of polycystin-2 is required for this interaction [37] (Fig. 1). The binding site was further identified as the acidic cluster in the C-terminus of polycystin-2, which interacts with a cluster of basic residues in the N-terminal suppressor domain of the IP_3_R [38]. Disruption of this molecular interaction by using competitive peptides eliminated the stimulation of IP_3_-induced Ca^2+^ release (IICR) by polycystin-2. In both studies, the channel death mutant (D511 V) did not provoke stimulation of IICR, which is a strong indication that polycystin-2 operates as a CICR channel that becomes activated by IICR in the immediate proximity of the IP_3_R-channel pore. Activation appears to be restricted to a microdomain of IICR as reduction of this microdomain by the fast Ca^2+^ buffer BAPTA eliminated activation of the CICR via polycystin-2, whereas the slower Ca^2+^ buffer EGTA did not have such effect [38]. It is conceivable that ER-localized polycystin-2 is silent in resting conditions as inappropriate opening of this channel would represent a Ca^2+^ leak from the ER eventually resulting in ER depletion and an ER-stress response [84]. At variance with these data, it was observed that exogenous expression of polycystin-2 in HeLa cells increased the ER Ca^2+^ permeability, which resulted in lowering of the ER Ca^2+^ content and a decrease in the histamine-evoked Ca^2+^ response [85]. The IP_3_R was not required for the polycystin-2-mediated reduction of the ER Ca^2+^ load, which suggested that polycystin-2 forms an independent ER Ca^2+^-leak channel [85]. The apparently contradictory results regarding amplification of IICR as a result of CICR via polycystin-2 [37, 38, 58], versus a diminished IICR due to ER Ca^2+^-store depletion [85], can possibly be reconciled by the cell- and condition-specific factors that regulate activation of polycystin-2 in the ER. Syntaxin-5, an ER- and Golgi-associated t-SNARE that functions in vesicle targeting and fusion, was found to directly interact with polycystin-2 and to inactivate its channel activity [86]. Syntaxin-5 was proposed to have a function in preventing the Ca^2+^ leak from the ER via polycystin-2. LLC-PK_1_ cells expressing Δ(5–72) polycystin-2 that lacks the syntaxin-5-binding site had a reduced ER Ca^2+^ content and a concomitant lower increase in cytosolic [Ca^2+^] upon agonist stimulation [86]. Polycystin-2 in the ER may therefore play an important role for controlling ER Ca^2+^ levels and its activity may be tightly controlled. Among the other polycystin-2-interacting proteins, polycystin-1 [87], α-actinin [88], mammalian diaphanous-related forming 1 (mDIA-1) [89], and fibrocystin [90] have been found to modulate polycystin-2′s activity.

Polycystin-2 was also found to bind and regulate the RyR2 in the sarcoplasmic reticulum (SR) of cardiac myocytes [39]. The C-terminus of polycystin-2 functionally inhibited the RyR2 channel and polycystin-2-deficient cardiomyocytes showed changes in store content and Ca^2+^-release properties. This altered RyR2 function could play a role in the development of cardiovascular abnormalities in ADPKD patients [39].

On the one hand, polycystin-2 can act as a regulator of other ER/SR channels, but on the other hand, it has its own channel properties that may be controlled by different cellular parameters. An obvious candidate for the regulation of polycystin-2′s channel properties in the ER is polycystin-1. As stated above, models were proposed for interaction of polycystin-1 in the plasma membrane with polycystin-2 in the ER [47, 81]. Although it is still unclear what the function could be of polycystin-1 at the level of the ER, there is compelling evidence that a significant amount of polycystin-1 is also localized there [43, 91]. This is particularly the case for shorter C-terminal cleavage forms, which were shown to functionally interact with the IP_3_R thereby inhibiting IICR [40]. The interaction site was found to be the IP_3_R ligand-binding domain similarly to the binding site for polycystin-2 but with an opposite effect. It was therefore proposed that polycystin-1 and polycystin-2 both interact with the IP_3_R in a complementary way to maintain a balance of proper ER-mediated Ca^2+^ signaling [40]. A different result was obtained by other authors [92], showing that exogenous expression of polycystin-1 in MDCK cells accelerated the decay of ligand-activated cytoplasmic Ca^2+^ transients. These data were interpreted as due to an inhibition of the Ca^2+^ leak across the ER membrane. These studies clearly illustrate the importance of polycystins in maintaining proper ER Ca^2+^ homeostasis [93]. Another unexpected finding, consistent with the reduction of the intracellular Ca^2+^ responses, was the identification of a P100 C-terminal fragment of polycystin-1 that is localized in the ER and interacts with STIM1 [41]. P100 probably contains the six C-terminal transmembrane domains and the C-terminal tail. It would interact with STIM1 via its coiled-coil domain and thereby interfere with the translocation of STIM1 to the plasma membrane, inhibiting the activation of store-operated Ca^2+^ currents. This would then lead to decreased store filling and subsequently to diminished agonist-induced Ca^2+^ transients [41]. It should be noted, however, that the expression of the C-terminal tail of polycystin-1 as a fusion protein yielded an opposite result and led to an increase in the cytosolic Ca^2+^ concentration and store-operated Ca^2+^ entry (SOCE) [94, 95]. This discrepancy may be due to a dominant negative effect of the polycystin-1 C-terminus, given the observation that the formation of P100 actually requires the presence of full-size polycystin-1 [41]. The nature of the polycystin-1 cleavage responsible for P100 generation and the significance of this mechanism are as yet unknown, but these findings clearly illustrate the importance of polycystin-1 as well as polycystin-2 for ER Ca^2+^ signaling. Mechanistically, polycystin-1 was proposed to increase the interaction between the IP_3_R and STIM1, which thereby inhibited Ca^2+^ release and SOCE [96] (Fig. 1). This regulation implicated the activation of the phosphatidylinositol 3-kinase (PI3-K)/protein kinase B (AKT) signaling pathway and would act upon a protein complex involving polycystin-2/IP_3_R/STIM1. It should be pointed out, however, that in a study comparing STIM1^−/−^ and wild-type MEF cells, no evidence was found for a direct interaction between STIM1 and the IP_3_R, but the effects on IICR were attributed to changes in the connections between the ER and plasma membrane [97].

The effects of polycystin-1 on ER Ca^2+^ release were mostly obtained by exogenous expression of polycystin-1 or its C-terminal fragments. The properties of endogenous polycystin-1 as obtained from knock-down experiments are much less documented. In studies of polycystin-1 haploinsufficiency in renal cells [98] or in vascular cells [99], the results pointed to a decreased resting cytosolic Ca^2+^ concentration in the polycystin-1^+/−^ as compared to the wild-type cells. Moreover, vascular smooth-muscle cells from polycystin-1^+/−^ mice exhibited a decreased agonist-induced Ca^2+^ release as compared to the wild type [99]. This result is in contrast to the data obtained with exogenous polycystin-1 expression, but it is more in line with the general concept that polycystin-1 and polycystin-2 form a functional complex with Ca^2+^-channel properties. Disturbance of this complex by either polycystin-2 or polycystin-1 knock-out is then expected to result in a decreased Ca^2+^-release activity. This is exactly what was recently found for the effect on IICR in a model system of plasma membrane-permeabilized cells upon lentiviral knock-down of either or both polycystins [100]. The presence of both polycystins seemed to be required for stimulation of Ca^2+^ release from the ER and knock-down of either polycystin decreased the apparent sensitivity of IICR [100]. The conflicting results from different groups may be caused by the fact that on the one hand exogenous expression may result in abnormal processing, trafficking and localization, and on the other hand knock-out or knock-down of endogenous genes may result in adaptive responses. For both approaches, the unraveling of the detailed mechanism is presently not at hand, but it becomes increasingly clear that both polycystins are implicated in ER-related Ca^2+^ fluxes. An interesting view that was presented in a model by the Guggino group [96], proposed a role for polycystin-1 in preventing a phenotype with more IICR and SOCE-mediated Ca^2+^ fluxes and promoting a situation with more filled stores and inactivated SOCE with Ca^2+^ influx via plasma membrane (or cilia)-localized polycystin-2 (Fig. 1). While not all data are easily reconciled with this model, it presents the very appealing idea that defective polycystins in ADPKD provoke a shift in the spatial properties of intracellular Ca^2+^ signals with the appearance of different microdomains with altered cytosolic Ca^2+^ concentrations, which can then elicit different downstream effects.

## Downstream effects of Ca^2+^ signaling in ADPKD

### Effects on cAMP signaling

Disturbed Ca^2+^ signaling is a proximal event in ADPKD and it directly or indirectly affects several other very important signaling pathways [101]. Among these, an increased cAMP concentration is a common finding in different models of ADPKD and cAMP stimulates cyst fluid and electrolyte secretion [102], possibly involving the stimulation of the cystic fibrosis transmembrane conductance regulator (CFTR) [103]. The reasons for the high cytosolic cAMP concentration in cyst cells are not very well understood. The polycystin proteins may alter the activity of G-protein-coupled receptors, like the V2 vasopressin receptor, that signal via cAMP [102, 104]. A link with cellular Ca^2+^ homeostasis was proposed via the activity of Ca^2+^-inhibitable adenylyl cyclase (AC) and/or Ca^2+^-dependent phosphodiesterase (PDE) [101]. The interaction between cytosolic Ca^2+^ and cAMP is, however, very complex, as there are nine different genes encoding transmembrane-domain ACs (AC1–9) along with numerous splice variants of the gene encoding soluble ACs, while also the cyclic nucleotide-PDE superfamily consists of over 30 genes [105]. Moreover, different members of these families are sensitive to Ca^2+^, either directly or via calmodulin. A common theme in the regulation of cAMP production is the pronounced intracellular compartmentalization. Three modes of cAMP microdomain/compartment formation are found: via (a) lipid rafts, (b) A-kinase anchor proteins (AKAPs), and (c) targeting of soluble AC to cellular organelles [105]. It can therefore be anticipated that a relation between polycystin dysfunction and the cAMP levels in ADPKD may be very much linked to specific microdomains either at the plasma membrane or at intracellular membranes and may involve particular AC or PDE isoforms. In this respect, it was recently demonstrated that the primary cilium plays an important role as a subcellular cAMP-signaling compartment [106]. A protein complex comprising AKAP150, AC5/6, and protein kinase A (PKA) was found in primary cilia of renal epithelial cells. Polycystin-2 and PDE4C were further identified as components of this ciliary AKAP complex. Under normal conditions PDE4C would promote the hydrolysis of cAMP and polycystin-2 could provide local accumulation of Ca^2+^ that inhibits the Ca^2+^-sensitive AC5 and AC6. Malfunction of polycystin-2 as a Ca^2+^ channel may reduce the local Ca^2+^ concentration in the cilium and thereby activate AC5/6. Other mechanisms like mutations of the transcription factor hepatocyte nuclear factor-1ß (HNF-1ß) were found to inhibit the expression of PDE4C and thereby increase cAMP levels [106]. As HNF-1ß also regulates the expression of *Pkd2* itself [107], downregulation of polycystin-2 and subsequent impaired ciliary trafficking of the AKAP complex may also contribute to the elevation of cAMP levels [106]. PDE1 isoforms (PDE1a, PDE1b, and PDE1c) are expressed to high levels in the kidney cells [108]. Importantly, these PDE isoforms are also regulated by cytosolic Ca^2+^, which in ADPKD would result in decreased PDE activity and a higher cAMP concentration [42]. An additional mechanism that could be important in ADPKD is cAMP production related to Ca^2+^-store depletion and STIM1 translocation [109]. These data are indicative of a mechanism of store-operated cAMP signaling, in which lowering of the Ca^2+^ concentration in the ER led to recruitment of ACs through a process involving STIM1 [109]. This mechanism was also found in polycystin-2-defective cholangiocytes, where polycystin-2 was suggested to play a role in SOCE activation and in inhibiting the STIM-dependent activation of AC6 [110]. In view of the observation that polycystin-1 expression impairs translocation of STIM to the plasma membrane, this mechanism could also link polycystin-1 defects to increased cAMP signaling in ADPKD [41, 96].

### Effects on B-Raf-extracellular signal-regulated kinase (ERK) signaling and cell proliferation (Fig. 2)

In general, cystic epithelia in ADPKD have high levels of cAMP and also of mitogen-activated protein kinase (MAPK) activity. This signaling pathway, involving cAMP-dependent PKA, stimulates the proliferation of cells from ADPKD cysts, but not cells from normal human kidney [111–113]. In normal cells, cAMP via PKA-mediated phosphorylation inhibits the MAPK pathway by blocking the activation of Raf-1 (Raf-C). In cystic cells, however, cAMP signaling is changed, an effect which is attributed to the low cytosolic Ca^2+^ concentration [114, 115]. Ca^2+^ restriction was associated with an elevation in B-Raf protein levels. In these conditions, cAMP stimulates B-Raf/MEK/ERK signaling in a sarcoma (Src)- and Ras-dependent manner. Moreover, the activity of AKT, a negative regulator of B-Raf, was decreased by Ca^2+^ restriction. Inhibition of AKT or PI3-K also allowed cAMP-dependent activation of B-Raf and ERK at normal Ca^2+^ levels. These results suggest that Ca^2+^ restriction causes an inhibition of the PI3-K/AKT pathway, which relieves the inhibition of B-Raf to allow the cAMP growth-stimulated phenotypic switch [112, 114]. The steady-state cytosolic Ca^2+^ concentration was found to be 20 nM lower in cyst-derived ADPKD cells compared with normal cells. Elevation of the cytosolic Ca^2+^ concentration in ADPKD cells increased AKT activity and blocked cAMP-dependent B-Raf and ERK activation. Thus, an increase in the cytosolic Ca^2+^ concentration was able to restore the normal anti-mitogenic response to cAMP [115], while Ca^2+^-channel inhibition by verapamil accelerated polycystic kidney-disease progression [116]. On the other hand, it was found that down-regulation of polycystin-1 using RNA interference [117] or expression of the dominant-negative polycystin-1 C-terminus [95, 118] resulted in an increase in basal Ca^2+^ concentration. The differential effects of polycystin-1 expression on the cytosolic Ca^2+^ concentration and downstream effects could reflect the use of different cell types such as immortalized cells versus primary cells [117].

**Fig. 2:**
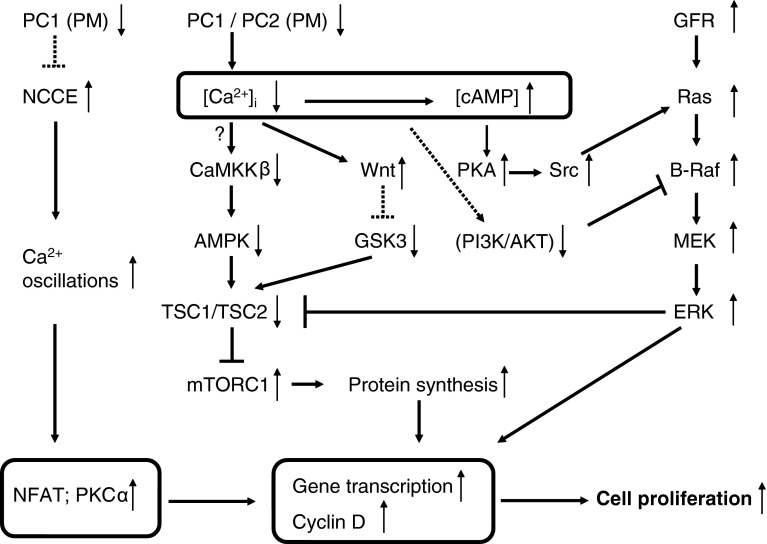
Signaling pathways that relate disturbed polycystin-mediated Ca^2+^ signaling to cell proliferation. Defects in polycystin functions generally lead to a decrease in the cytosolic Ca^2+^ concentration ([Ca^2+^]_i_). This results in an increase in cAMP concentration, probably via changes in Ca^2+^-dependent phosphodiesterase or Ca^2+^-dependent adenylate-cyclase activity (not shown). The Ca^2+^-cAMP link is represented by the *box*. In the presence of low [Ca^2+^]_i_, cAMP becomes pro-proliferative via activation of the Src/Ras/B-Raf/MEK/ERK pathway. Ca^2+^ restriction causes decreased PI3-K/AKT signaling, which relieves the inhibition of B-Raf. Activation of growth-factor receptors with tyrosine-kinase activity (GFR) also contributes to the stimulation of MAPK/ERK signaling and cell proliferation. Polycystin-1 dysfunction thus upregulates the MAPK/ERK pathway, which results in inactivation of the tuberin complex and increased mTORC1. Cytoplasmic Ca^2+^ also regulates the mTOR pathway via CaMKKß and AMPK (the *question mark* indicates that the occurrence of this mechanism was not yet explored in cystic cells). The deregulated Ca^2+^ signaling switches on canonical Wnt signaling, which activates mTOR via inhibiting GSK3 phosphorylation of tuberin. Another link to cell proliferation may depend on the activation of Ca^2+^ oscillations and subsequent effects on gene transcription and cyclins via Ca^2+^-dependent NFAT or via protein kinase Cα (PKCα) signaling. *Boxed areas* indicate mechanisms not shown in detail. *Dotted arrows* indicate still-unresolved mechanisms. *GFR* growth-factor receptor

### Effects on mammalian target of rapamycin (mTOR) signaling (Fig. 2)

Cyst-lining epithelial cells exhibit higher levels of mTOR signaling as compared to normal epithelial cells [119]. mTOR is a serine/threonine protein kinase that provides catalytic activity for two distinct multiprotein complexes (mTORC1 and mTORC2). mTORC1 is a metabolic sensor and its activation promotes cell growth and cell proliferation. The effects of mTORC2 include modulation of cell survival, cytoskeletal organization, and cell polarity [120]. Hyperactivity of mTORC1 and possibly also of mTORC2, contributes to cyst formation [121]. Tuberin, the protein product of *Tsc2*, in a complex with hamartin, the product of *Tsc1*, is the primary negative regulator of mTORC1. Tuberin is a GTPase-activating protein (GAP), which regulates Rheb, a small G-protein belonging to the Ras superfamily. Hamartin and tuberin form a heterodimer, which converts Rheb-GTP to the inactive Rheb-GDP. Rheb is an activator of mTORC1 and conversion of Rheb to the GDP-bond form inactivates the mTORC1 pathway [120–123]. The tuberin–hamartin complex is a nodal point for several polycystin-dependent pathways. The cytoplasmic tail of polycystin-1 interacts with tuberin. This may occur via direct interaction with tuberin [119] or indirectly via ERK-dependent phosphorylation and inactivation of the tuberin complex [124]. A prominent activation of the ERK pathway together with cystogenesis was found upon inactivation of the *Pkd1* gene in conditional knock-out mice [125]. The regulation may, however, be more complex as tuberin has been reported to be phosphorylated by at least nine distinct protein kinases [126]. Among these are multiple pathways that may be linked to proper functioning of polycystins and some of these are clearly linked to Ca^2+^ (Fig. 2). As explained above, a potential link with Ca^2+^ and cAMP may occur via B-Raf/ERK signaling. The lower Ca^2+^ levels in cystic cells would together with the increased cAMP concentration lead to activation of B-Raf/ERK and subsequent mTORC1 signaling [42, 114, 115]. Another mechanistic input of polycystins on mTORC1 is via ciliar activation of liver kinase B1 (LKB1), which, like the polycystins, is localized in the basal bodies of primary cilia [127]. LKB1 activates AMP-activated protein kinase (AMPK), which then activates tuberin with subsequent negative regulation of mTORC1. This cilium-mediated effect on mTOR was, however, found to be independent of flow-induced Ca^2+^ transients or AKT [127]. Next to LKB1, however, AMPK activity is also directly linked to the cytosolic Ca^2+^ concentration by calmodulin-dependent protein kinase kinase ß (CaMKKβ) [128]. The link between the changed cytosolic Ca^2+^ concentration in cystic cells and mTORC1 via CaMKKβ-dependent regulation of AMPK is, however, not yet established. A relation between AMPK activity and cyst formation was, however, found by using metformin, a drug in wide clinical use as a pharmacological activator of AMPK [129]. Stimulation of AMPK activity by metformin resulted in inhibition of the mTOR pathway and significant arrest of cystic growth in in vitro and ex vivo models of renal cystogenesis [129].

Additional pathways may converge on mTOR signaling in a Ca^2+^-dependent way. Wnt signaling, which was shown to be regulated by polycystin-1 [130], can activate a ß-catenin-dependent (canonical) and a ß-catenin-independent (non-canonical) pathway. In addition, a Wnt-Ca^2+^ pathway influences both canonical and non-canonical pathways [131]. Inversin, a ciliar protein, functions as a molecular switch between the different Wnt signaling pathways [132]. It can be speculated that ciliary events as flow-induced Ca^2+^ influx can switch off the canonical and activate the non-canonical pathway [42]. Over-activation of canonical Wnt and subsequent over-production of activated ß-catenin would promote the polycystic phenotype [133]. This is further supported by many observations showing a link between disturbed polycystin function and enhanced activity of Wnt signaling [130, 134–136]. In addition, the Wnt pathway is also able to act on mTOR via AMPK and GSK3 [137] (Fig. 2). Tuberin was reported to be a physiological substrate of GSK3, which required priming phosphorylation by AMPK. Canonical Wnt then activates mTOR via inhibiting GSK3 phosphorylation of tuberin [137]. Moreover, GSK3ß was shown to be activated by polycystin-1 [138]. In ADPKD, disturbed polycystin-1 function and disturbed Ca^2+^ signaling may thus both contribute to a GSK3-dependent increase in mTOR activity [124].

### Effects on cell proliferation and apoptosis (Figs. 2, 3)

Kidneys from ADPKD patients have high levels of apoptosis in addition to increased cellular proliferation [139–141]. This dysregulation of both apoptosis and proliferation may represent a general mechanism for cyst growth and remodeling [140, 142], and the imbalance between pro-apoptotic and pro-proliferative factors was proposed to be critical for the development of cystic kidney disease [143–145]. Different signaling pathways may be implicated in the abnormal cell-cycle progression. Polycystin-1 activates the JAK-STAT pathway, thereby up-regulating p21 (waf1), an inhibitor of cyclin-dependent kinase (CDK), and thereby inducing cell-cycle arrest in G_0_/G_1_. This process requires polycystin-2 as an essential cofactor [146]. A decrement of p21 in cystic kidneys as compared to non-cystic kidneys was demonstrated in humans and rat models [147]. Roscovitine, which has been shown to arrest progression in a murine model of polycystic kidney disease, increases p21 levels and decreases renal tubular epithelial-cell proliferation [147, 148]. Renal tubular epithelial cells exposed to “low” concentrations of roscovitine showed minimal apoptosis in association with markedly increased levels of the anti-apoptotic protein p21, and these cells became senescent. Conversely, cells exposed to “high” levels of roscovitine became apoptotic [149]. Tubular-cell apoptosis occurs in most animal models of ADPKD like the SBM mouse [150, 151] and the Han:SPRD rat model [152], as well as in kidneys from ADPKD patients [139–141]. Mice deficient in the anti-apoptotic Bcl-2 gene develop polycystic kidney disease characterized by dilated proximal and distal tubular segments and hyper-proliferation of the epithelium and interstitium [153, 154]. While ablation of the pro-apoptotic Bim prevented the development of polycystic kidney disease in mice deficient in Bcl-2 [155], this was not the case in polycystin-1-deficient mice. This indicates that loss of Bcl-2 or loss of polycystin-1 elicit polycystic kidney disease through different mechanisms [156]. Similarly, deletion of another anti-apoptotic gene, the AP2ß transcription factor, in AP2ß^−/−^ mouse resulted in polycystic kidney disease with concomitant down-regulation of anti-apoptotic Bcl-2-family proteins and massive apoptotic cell death [157]. A direct cause-and-effect relationship between cyst formation and apoptosis was demonstrated in Han:SPRD rats using caspase inhibitors. Caspase inhibition was found to reduce tubular apoptosis and proliferation and to slow disease progression in polycystic kidney disease [158]. A marked increase in caspase-3 and -7 activity has been reported in the Han:SPRD rat [143, 159, 160], and targeted caspase-3-gene deletion prolongs survival [161]. In the SBM mouse that overexpresses the proto-oncogen c-myc, both proliferative and apoptotic indexes were highly increased, reflecting a critical imbalance in c-myc regulation of the opposing processes of cell proliferation and apoptosis [162]. Overexpression of c-myc was found in cystic tissue and is supposed to play a role in the dysregulation of both proliferation and apoptosis in ADPKD [140, 162–165].

**Fig. 3:**
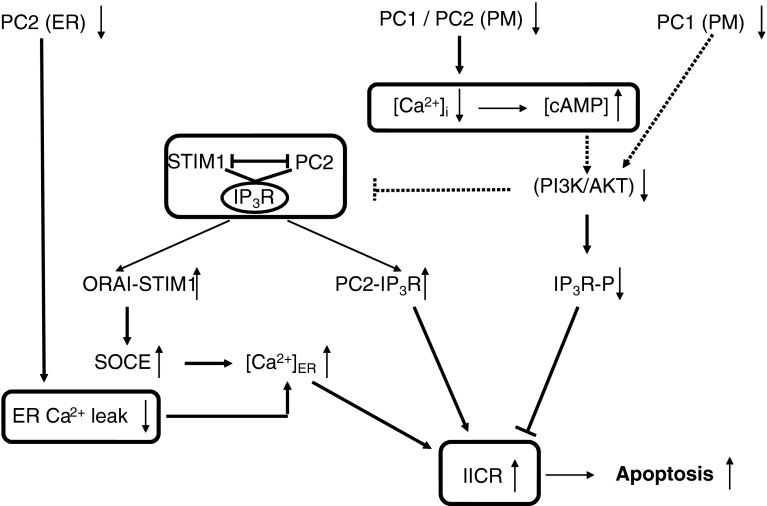
Signaling pathways relating disturbed polycystin-mediated Ca^2+^ signaling to apoptotic cell death. Disruption of PC1 function leads to a phenotype with low intracellular Ca^2+^ concentration and high cAMP concentration that showed a mitogenic response towards cAMP and down-regulation of PI3-K/AKT. This provokes a profound remodeling of the relation between Ca^2+^ release and Ca^2+^ influx via an IP_3_R/PC2/STIM1 protein complex. Decreased AKT signaling would strengthen the IP_3_R-PC2 interaction and lead to increased IICR, translocation of STIM1 to the plasma membrane, and refilling of the ER via SOCE. AKT can also directly phosphorylate the IP_3_R thereby inhibiting its activity. A decreased AKT activity in cystic cells would thereby relieve the inhibition of the IP_3_R and contribute to the increase in IICR. PC2 can function as an ER Ca^2+^-leak channel and a loss of function would therefore increase the ER Ca^2+^ content and IICR. The increased IICR via these different mechanisms ultimately leads to increased Ca^2+^ transfer from the ER to the mitochondria. Mitochondrial Ca^2+^ overload is a very important determinant of Ca^2+^-dependent apoptosis. *Boxed areas* indicate mechanisms not shown in detail. *Dotted arrows* indicate still unresolved mechanisms. IP_3_R-P: IP_3_R phosphorylated by AKT (S^2681^ in IP_3_R1)

Ca^2+^ signaling is implicated in the phenotypic feature of ADPKD cells showing elevated rates of both proliferation and apoptosis, but the downstream mechanisms are not fully resolved. It was found that polycystin-1 induces resistance to apoptosis and normal tubulogenesis through the PI3-K/AKT pathway [166] (Fig. 3). These data are consistent with observations that loss of polycystin-1 function results in changes in cytosolic Ca^2+^ concentration, down-regulation of PI3-K/AKT and activation of B-Raf/ERK in ADPKD cysts [114]. As already outlined above, the disturbed intracellular Ca^2+^ signaling could be a primary event in ADPKD and may be responsible for the switch to a proliferative phenotype with an elevation in B-Raf protein levels and cAMP-stimulated, Ras-dependent activation of B-Raf and ERK [114, 115]. Also, a *PKD2* transgenic mouse model resulted in renal-cys formation via B-Raf signaling, probably by acting as a dominant negative modulator for polycystin function and downstream Ca^2+^ signals [167]. *Pkd2* ^+/−^ vascular smooth-muscle cells also have an abnormal phenotype with a defective Ca^2+^ signaling with decreased levels of the cytosolic Ca^2+^ and an increased cellular cAMP concentration, which is probably underpinning increased proliferation and apoptosis [168]. The low cytosolic Ca^2+^ concentration apparently provoked increased proliferation in these vascular smooth-muscle cells but the effect in this cell type was independent of cAMP/B-Raf signaling [168].

A divergent mechanism was proposed, in which disturbed polycystin-1 function affected cell proliferation by an increase in intracellular Ca^2+^ signaling in the form of activation of Ca^2+^ oscillations [117] (Fig. 2). Serum treatment of HEK293 cells with down-regulated polycystin-1 or of cystic cells expressing mutated polycystin-1 resulted in increased oscillatory activity of the cytosolic Ca^2+^ concentration which led to activation of cell proliferation. The proposed mechanism involved non-capacitative Ca^2+^ entry (NCCE), which was proposed to be negatively regulated by polycystin-1. The loss of polycystin-1 resulted in NCCE activation and increased Ca^2+^ oscillations. The downstream effects were related to activation of the calcineurin/nuclear factor of activated T cells (NFAT) pathway. The explanation could be that Ca^2+^ oscillations rather than the basal Ca^2+^ concentration are crucial for activation of the NFAT-dependent cell proliferation [117].

Another important factor, particularly for the increased apoptosis in ADPKD, could be the regulation of the ER Ca^2+^ content by polycystins [84] (Fig. 3). The ER Ca^2+^ content is determined by the activity of Ca^2+^ pumps and by the Ca^2+^-leak rate via different pathways. The resulting ER Ca^2+^ load is a primary determinant of the extent of ER-to-mitochondrial Ca^2+^ transfer and pro-apoptotic Ca^2+^ signaling [84]. Polycystin-2 in the ER may act as a Ca^2+^-leak pathway and in this way control the degree of ER Ca^2+^ filling [85]. Normal polycystin-2 functioning would then reduce Ca^2+^ release from the ER in response to apoptotic stimuli, and conversely, its loss in ADPKD would lead to an increased apoptosis [85]. Although this mechanism may provide a simple molecular explanation for the increased apoptosis rate in ADPKD upon loss of polycystin-2 function, the situation may be more complex. As discussed above, polycystin-2 activity and its modulation of ER Ca^2+^ are probably tightly regulated by many other cellular factors and interacting proteins. Not only is polycystin-2 a Ca^2+^ channel but it also interacts with the two main intracellular Ca^2+^-release channels, IP_3_Rs and RyRs, and with various plasma-membrane TRP channels. Polycystin-1 controls the activity of polycystin-2 directly and also in an indirect way via PI3-K/AKT signaling. Decreased PI3-K/AKT signaling in ADPKD would thereby lead to a profound remodeling with increased IICR and SOCE (Fig. 3). This results from activation of Ca^2+^ release via polycystin-2, but also from a higher IP_3_R activity resulting from the relieve of the brake imposed by AKT-mediated phosphorylation [169]. Increased IICR, particularly at the contact sites of the ER and mitochondria, constitutes a strong apoptotic signal [84, 170].

In conclusion, disturbed or remodeled cellular Ca^2+^ signaling is clearly a very early event in the development of the cystic phenotype of renal cells. This phenotype is characterized by a concomitant activation of both cell proliferation and apoptotic cell death. Despite the increased proliferation as an invariable component of cystogenesis, there is seldom progression towards renal carcinoma [171]. Overexpression of polycystin-1 provoked apoptosis in different cancer cell lines, and it is tempting to speculate that polycystins, by controlling the balance between proliferation and cell death, may play a role in preventing malignant transformation [171]. ADPKD epithelial cells are thereby characterized by a modest degree of cell proliferation together with a proportional increase in apoptosis [139, 140, 146, 153, 157, 172]. Both polycystins are closely involved in cellular Ca^2+^ signaling by direct or indirect interaction with many proteins of the cellular Ca^2+^ toolkit. It becomes increasingly evident that polycystin dysfunctions lead to a profound remodeling of cellular Ca^2+^ signaling and provoke changes in the spatio-temporal modes of Ca^2+^ signaling. This could lead to changes in the occurrence of different Ca^2+^-signaling microdomains located at the cilia or at the ER-mitochondria junctions, and it could lead to oscillatory Ca^2+^ signals that may evoke nuclear responses. It will be a challenge for future research to experimentally detect the subcellular Ca^2+^-signaling microdomains and to identify their downstream responses that result in the increased proliferation and increased apoptosis responses, which are characteristic for the cystic phenotype.
